# Africa cannot afford to wait for malaria treatment failure

**DOI:** 10.5281/zenodo.19825249

**Published:** 2026-05-02

**Authors:** Deepa Pindolia, Rosario Martinez-Vega, Gulnaz Uzakbayeva, Deus Ishengoma, Olivia Ngou, Roly Gosling

**Affiliations:** 1 Independent consultant, Germany.; 2 London School of Hygiene and Tropical Medicine, UK.; 3 Ifakara Health Institute and National Institute for Medical Research, Tanzania.; 4 Impact Santé Afrique, Cameroon.; 5 Malaria Elimination Initiative, Institute of Global Health Science, University of California San Francisco, USA.

## Abstract

For more than two decades, artemisinin-based combination therapies (ACTs) have been the backbone of malaria treatment across Africa. They have saved millions of lives and averted many malaria cases. That foundation is now under threat. Artemisinin partial resistance (ART-R) is now being detected using molecular surveillance across eastern Africa, the Horn of Africa, and southern Africa. It has already been confirmed in four countries: Rwanda, Uganda, Eritrea and Tanzania, with signals reported in several neighbouring countries [1].

This isn't treatment failure yet, but it's what comes before it. Antimalarial drug resistance follows a predictable pattern: early warning signals appear, they spread, and then widespread treatment failure follows. That's how artemisinin resistance unfolded in Southeast Asia [2]. Africa is now on a similar path, with far higher stakes. When treatment starts to fail, patients stay sick longer, transmission rises, and already overstretched health systems struggle to cope.

## The risk is no longer theoretical

**Figure 1:**
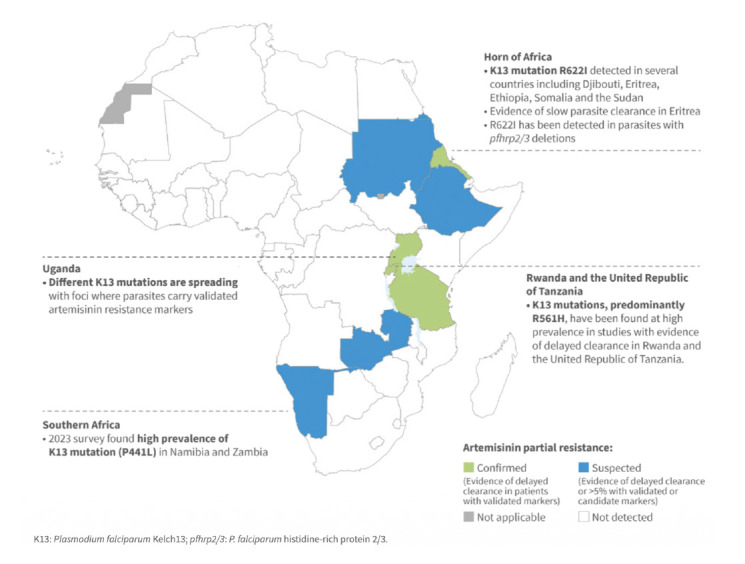
Map of artemisinin partial resistance in Africa (Source: World Malaria Report 2025).

ART-R (Figure 1) has emerged independently in multiple locations across Africa [3], rather than spreading from a single origin. At the same time, there are already signs of declining efficacy of artemether–lumefantrine, the most widely used ACT globally, in multiple settings, including Angola, the Democratic Republic of Congo, Uganda and Tanzania [4-7]. If not addressed early, this can lead to broader treatment failure, with serious consequences for malaria control and already stretched health systems..

## We are seeing the problem but not acting on it

**Figure 2:**
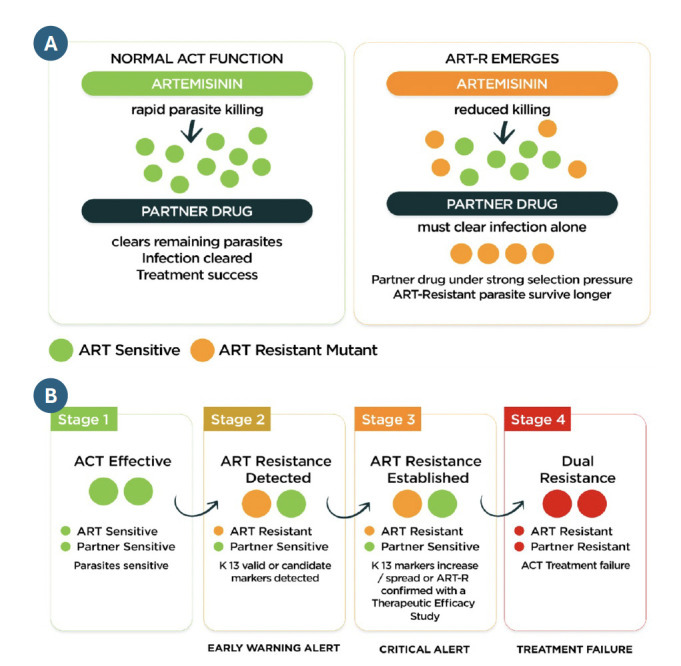
Moving from detection to action: Combining data to enable earlier, targeted responses to antimalarial drug resistance. A: Artemisinin shifts selection pressure. B: Drug resistance causes ACT failure. Source: AMDR Action [10].

Across much of the continent, surveillance systems are doing what they are supposed to do. They are detecting ART-R mutations in malaria parasites, the early warning signals of antimalarial drug resistance, and identifying the areas where resistance is emerging [8]. The real challenge is what happens next. These signals are not consistently triggering action. Too often, the response stops at detection. Countries are left with data, but without the financing, coordination, or readiness to act on it. Readiness for treatment policy change remains limited, and responses are often fragmented or delayed. At the same time, major financing and planning cycles—such as Global Fund Grant Cycle 8 (GC8)—are underway, yet in many settings ART-R has not been fully translated into concrete programmatic shifts or investment priorities. The result is a clear disconnect: The risk is visible, but action is lagging. Figure 2 illustrates how available data can be used to trigger earlier action, rather than stopping at detection.

## What needs to change

Many of the actions needed are already known and can be implemented now. A set of ‘no-regret actions’ has been defined: Practical steps that countries can take immediately, even before additional funding is mobilised. These include acting on early warning signals in hotspot areas, changing treatment policy in the places with ART-R and neighbouring areas, and strengthening coordination across borders. Regional platforms—such as those in the East African Community, the Horn of Africa, and southern Africa through mechanisms like SADC—provide a critical opportunity to align policies, coordinate responses, and act at the scale required to contain resistance. The full set of actions is available online [9]. This requires a paradigm shift: from waiting for treatment failure to acting on early warning signals.

## The window is closing

Each year of delay increases the risk that resistant parasites spread further, making future responses more complex and costly. The current moment, where funding decisions, regional collaboration, and technical guidance are all in motion, offers a narrow but real opportunity to act ahead of widespread failure. This window for early action is closing.

## This is bigger than malaria

If ACTs begin to lose effectiveness, the impact will go beyond malaria programmes. It will affect health system capacity, household costs, and economic productivity and raise broader health security concerns, including the risk of resistant parasites spreading beyond the continent. We have a limited variety of antimalarials, and once they fail, we are without obvious replacement.

## A moment to act

Africa is at a critical inflection point. Early, coordinated action can still protect the effectiveness of existing treatments and sustain progress made over the past two decades. Waiting for clear treatment failure will mean acting too late. The tools, data, and platforms to act already exist—what is needed now is to use them decisively. The warning signs are already clear. The question is whether we respond to them.
